# Effectiveness of Baby-Friendly Hospital Initiative on Early Initiation and Exclusive Breastfeeding Practice: Systematic Review and Meta-Analysis

**DOI:** 10.3390/nu17142283

**Published:** 2025-07-10

**Authors:** Mahilet Berhanu Habte, Misra Abdulahi, Michelle Plusquin, Charlotte Cosemans

**Affiliations:** 1 Department of Population and Family Health, Faculty of Public Health, Jimma University, Jimma P.O. Box 378, Ethiopia; 2 Centre for Environmental Sciences (CMK), Hasselt University, 3590 Diepenbeek, Belgiumcharlotte.cosemans@uhasselt.be (C.C.)

**Keywords:** baby-friendly hospital initiative, early initiation, exclusive breastfeeding, systematic review, meta-analysis

## Abstract

**Background**: The Baby-Friendly Hospital Initiative (BFHI) promotes, protects, and supports optimal breastfeeding through facility-based strategies. While prior studies have examined individual BFHI components in specific contexts, global evidence on its overall impact remains limited. This systematic review and meta-analysis aimed to evaluate the BFHI’s effectiveness in improving early initiation and exclusive breastfeeding practices worldwide. **Methods**: A comprehensive search was conducted in PubMed, Web of Science, Scopus, and Google for English-language studies. Eligible studies included randomized controlled trials (RCTs), cluster RCTs, and quasi-experimental designs assessing BFHI’s effect on breastfeeding outcomes. Random-effects meta-analysis models were used to estimate the pooled effects with 95% confidence intervals (CI). Heterogeneity was assessed using *I*^2^ statistics and *p*-values. Study quality was appraised using the GRADE approach. **Results**: Thirty studies met the inclusion criteria. The BFHI was associated with increased early initiation of breastfeeding (pooled RR 1.43; 95% CI: 1.12–1.81; *I*^2^ = 97.1%). Positive associations were also observed for exclusive breastfeeding at four months (RR 1.18, 95% CI: 1.08–1.29; *I*^2^ = 61.7%) and at six months (RR 1.56, 95% CI: 1.14–2.14; *I*^2^ = 82.8%). Substantial heterogeneity reflected variability in study design, BFHI implementation fidelity, and context. **Conclusions**: Our findings suggest that the BFHI is effective in improving breastfeeding practices globally. However, study variability and partial implementation may limit the generalizability of results. High-quality RCTs assessing full BFHI implementation are needed to strengthen evidence and guide global maternal–child health policy.

## 1. Introduction

Early initiation of breastfeeding (EIBF) is the practice of immediate breastfeeding initiation within the first hour of birth. EIBF has numerous benefits for newborns and mothers. The skin-to-skin contact immediately after birth helps regulate the newborn’s body temperature. Colostrum, the yellowish or golden first milk produced in the first hour of birth, provides essential nutrition and immune protection, reducing neonatal infections and mortality [1]. EIBF enhances the duration of exclusive breastfeeding, stimulates milk production, strengthens maternal–infant bonding, and promotes early uterine contraction and placental expulsion, thereby reducing postpartum hemorrhage [2,3]. Furthermore, exclusive breastfeeding (EBF) protects against childhood infections, such as diarrhea, pneumonia, and otitis media [4]. At the same time, the long-term benefits include lowering risks of dental caries, obesity, and type 2 diabetes and improved cognitive development [5], as well as cellular adaptations, as breastfeeding has been associated with higher mitochondrial DNA content in adolescents [6]. Globally, scaling up optimal breastfeeding could save over 820,000 children under five years old annually, as non-breastfed infants face significantly higher mortality risks from infections and other causes [3,7].

The WHO and UNICEF launched the Baby-Friendly Hospital Initiative (BFHI) in 1991 to promote and protect maternal and child health by ensuring support for breastfeeding in maternity care health facilities [8]. It is also one target of the Global Strategy for Infant and Young Child Feeding [9]. The BFHI’s Ten steps to successful breastfeeding provide evidence-based guidelines for maternity facilities to support breastfeeding through changes to policy, training, and practice. Health facilities and healthcare workers are key to implementing the BFHI [2], and the WHO grants accreditation to health facilities only if they successfully implement all ten steps; otherwise, accreditation is not granted.

Despite these efforts, global breastfeeding rates remain suboptimal. In 2023, only 46% of mothers initiated breastfeeding within the first hour after birth, and only 48% of these exclusively breastfed [10]. Regional disparities persist, whereby exclusive breastfeeding rates at six months stand at 47% in Africa, 51% in Asia, and a concerning 13% in Europe [11,12,13]. According to the public health monitoring agency Growing Up in Flanders (Belgium), 82.6% of newborns were breastfed within 24 h after birth, 36.4% were still receiving breast milk at six months, and only one out of ten children received exclusive breastfeeding up to six months of age [14]. Achieving the 2030 global target of 70% coverage for both indicators requires urgent action [15].

Existing studies on the effectiveness of the BFHI report mixed findings. While studies in Mexico, Bangladesh, Spain, and Turkey show a significant improvement in breastfeeding practices in the BFHI implementation group compared to those who received routine care [16,17,18,19], studies conducted in Finland and Nigeria found no statistically significant differences [20,21].

A previous review provided an overview of interventions consisting of three or more BFHI steps and included observational studies [22]. However, to our knowledge, no studies have systematically evaluated whether the incremental addition of one, two, three, or more BFHI steps is associated with stronger effects on breastfeeding outcomes in intervention studies. Therefore, this systematic review and meta-analysis assesses the overall effectiveness of the BFHI compared to routine care on EIBF and EBF during the first six months of life among term infants. In addition, we conduct analyses to explore whether the number of implemented steps influences the strength of the association. By synthesizing the evidence, this review aims to summarize scientific evidence for the BFHI, guide future intervention studies, and accelerate progress toward international targets for breastfeeding practice.

## 2. Methods and Materials

### 2.1. Search Strategy

This systematic review followed the guidelines outlined in the Cochrane Handbook for Systematic Reviews of Interventions [23]. The review protocol was registered with PROSPERO (CRD42024521636). A comprehensive literature search was conducted in four major databases, including PubMed, Web of Science, Scopus, and Google Scholar (for gray literature). The initial search string was developed for PubMed (MEDLINE) and subsequently adapted for the other databases. The search terms were based on four key concepts: (i) BFHI, (ii) EIBF, (iii) EBF, and (iv) infants aged 0–6 months. Citation tracking was also performed to maximize coverage.

This review followed the three-step search strategy recommended by the Joanna Briggs Institute (JBI). First, a limited search was conducted in PubMed to identify relevant keywords and index terms from the titles, abstracts, and full texts. Based on this, a refined and comprehensive search strategy was developed. The final search incorporated Medical Subject Headings (MESHs), entry terms, keywords, and relevant Boolean operators (AND, OR) across all databases (details on the search strategy can be found in the Supplementary Information).

**Figure 1:**
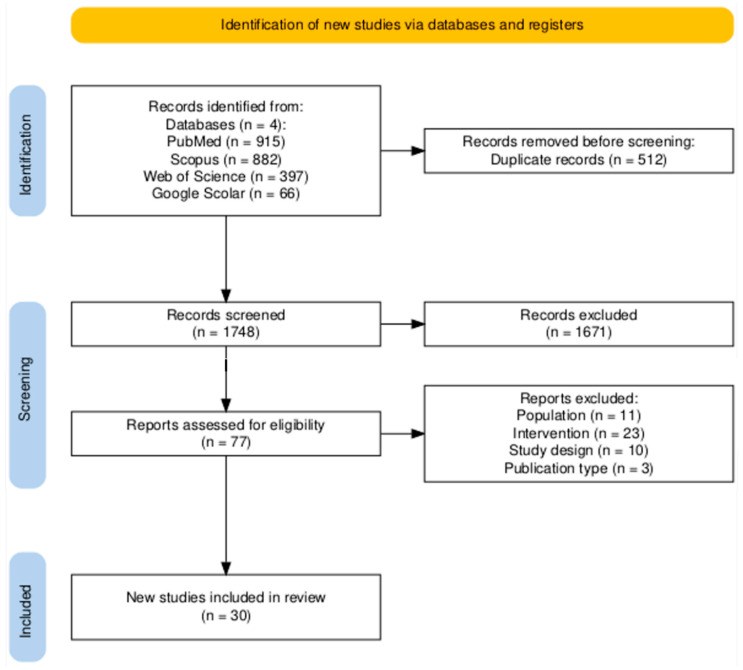
PRISMA flow diagram for study selection [26].

Additionally, a manual search of the reference lists of the included studies and relevant reviews was also performed to identify additional articles. Searches were limited to studies published in English from the start of BFHI intervention until 1 April 2025, as well as randomized controlled trials (RCTs), cluster RCTs, and quasi-experimental studies assessing the effect of the BFHI on breastfeeding outcomes. All retrieved records were imported into EndNote version 21 to remove duplicates, and the remaining references were imported into Rayyan software [24] for screening. Title and abstract screening was followed by full-text reviews to assess studies against the inclusion criteria. Any disagreements regarding study eligibility were resolved through discussion and consensus among reviewers. The review findings were reported following the Preferred Reporting Items for Systematic Reviews and Meta-Analyses (PRISMA) 2020 guidelines [25] (Figure 1).

### 2.2. Eligibility Criteria

Studies were eligible for inclusion if they evaluated the effectiveness of implementing at least one of the BFHI steps in improving EIBF within the first hour of birth and/or reporting EBF, including data on its duration. Eligible studies involved mother–newborn pairs where delivery occurred in a hospital setting without complications, resulting in a single, healthy newborn. The review included individual RCTs, cluster RCTs, and quasi-experimental studies. Only articles published in English were considered. The exclusion criteria included studies involving mother–newborn pairs transferred from other facilities or home settings, mothers with medical contraindications to breastfeeding, and mothers living with HIV.

### 2.3. Outcome

This review focused on two primary outcomes: EIBF and EBF practices. EIBF was defined as the initiation of breastfeeding within one hour of birth, and EBF was based on mothers’ self-reports regarding their practice of feeding their infants only breast milk.

### 2.4. Data Extraction and Management

Two reviewers performed data extraction (M.B.H. and C.C.). Disagreements were resolved through discussion or by consulting a third reviewer. The extracted data included the author, publication year, country, study design, specific BFHI steps implemented, sample sizes of the intervention and control groups, and proportions of early initiation and exclusive breastfeeding practice in each group. A descriptive synthesis was performed to summarize the findings about the types of BFHI interventions and breastfeeding outcomes (the table in Section 3.5).

Meta-analyses were performed using R Studio version 4.4.1 with meta package version 8.0.1, and we employed random-effects models to account for between-study heterogeneity in intervention effects. Intervention differences, country, methodological differences (study design and risk of bias), and statistical diversity were considered to assess heterogeneity. Given that the outcomes were reported as proportions, unstandardized effect sizes were used. Heterogeneity was evaluated using a standard χ^2^ test and quantified with the *I*^2^ statistic. An *I*^2^ value between 75% and 100% indicates substantial heterogeneity. Subgroup analyses were performed to explore sources of heterogeneity, such as sensitivity analysis performed based on socio-economic status, the study design, and the duration of EBF practice. All results are reported with 95% confidence intervals and *p*-values.

### 2.5. Risk-of-Bias Assessment of Individual Studies

Two reviewers (M.B.H. and C.C.) independently assessed the risk of bias in each included study using the Cochrane Risk-of-Bias (RoB2) Tool (version 2) [27]. The tool evaluates the following five domains of bias: (i) bias arising from the randomization process; (ii) bias due to deviations from the intended intervention; (iii) bias due to missing outcome data; (iv) bias in the measurement of the outcome; and (v) bias in the selection of the reported result. For each domain, studies were rated as having a ‘low risk’, ‘some risk’, or a ‘high risk’ of bias (the table in Section 3.2).

## 3. Results

### 3.1. Study Selection

A total of 2260 potentially relevant records were identified through electronic database searches (Figure 1). After removing 512 duplicates, 1748 articles were screened by title and abstract, resulting in the exclusion of 1671 studies. The remaining 77 full-text articles were assessed for eligibility, of which 30 met the inclusion criteria for this systematic review. Among these, 17 studies evaluated exclusive breastfeeding practice at four and/or six months. Six studies assessed the early initiation of breastfeeding within the first hour of birth. The remaining studies reported exclusive breastfeeding at earlier time points (less than four months).

### 3.2. Study Characteristics

The 30 included studies encompassed a total of 15,059 newborns. The study characteristics are presented in Table 1. According to the World Bank classification based on gross national income per capita [28], 3 studies were conducted in low-income countries (Nigeria, Sudan, and Bangladesh), 12 in middle-income countries (China, Malaysia, India, Iran, Turkey, Mexico, Greece, and Brazil), and 15 in high-income countries (United Kingdom (UK), Finland, United States of America (USA), France, Spain, and Canada). Twenty studies were individual RCTs, three were cluster RCTs, and seven employed quasi-experimental designs. The sample sizes ranged from 40 to 2724 participants. The duration of interventions varied from one hour to six months.

### 3.3. Outcome Measure

Exclusive breastfeeding was defined as the infant receiving only breast milk with no additional food or drink. Twenty-seven studies reported exclusive breastfeeding at different time points, ranging from 24 h to six months. Only six studies reported the early initiation of breastfeeding within the first hour of birth.

### 3.4. Intervention Types

All studies investigated the effect of at least one step of the BFHI. Among the total of 30 included studies, 17 assessed the impact of step 10 with or without other steps, followed by step 3, which was assessed in 8 studies in combination with other steps or independently (Table 2). Further details on the BFHI steps are found in the Supplementary Materials.

### 3.5. Risk-of-Bias Assessment

Of the 30 included studies, eleven were assessed as having an overall low risk of bias, eleven had some risk of bias, and eight were judged to have a high risk of bias (Table 2). Eight studies had a high risk of bias related to the randomization process, and five studies had some risk of bias regarding deviations from the intended intervention. Seven studies had some concerns about bias due to missing data. All studies had a low risk of bias in measuring outcomes of interest and reporting.

### 3.6. BFHI and Exclusive Breastfeeding Practice

**Figure 2:**
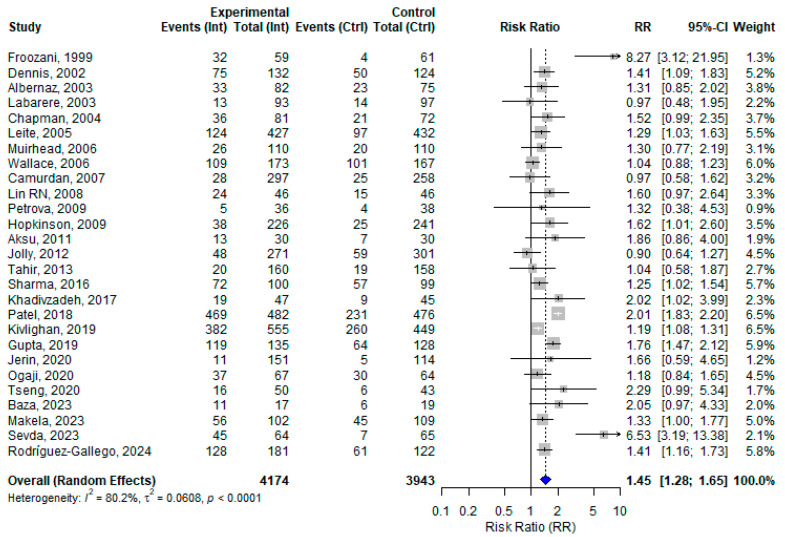
A random effects meta-analysis comparing the risk ratio of exclusive breastfeeding practice among mother–infant pairs at different follow-up period who received BFHI intervention and routine care. The blue diamond is the pooled estimate. The arrow indicates estimates greater than 10 (as the x-axis ends at 10) [16,17,18,19,20,21,29,30,31,32,33,34,35,36,38,39,40,41,42,43,44,45,46,47,48,50,51].

Among the 30 included studies, 27 assessed the effect of at least one BFHI step on EBF in different follow-up periods. The remaining three papers assessed only EIBF within the first hour of birth. From the 27 studies assessing EBF in various follow-up periods, the pooled effect size of BFHI interventions compared to routine care was 1.45 (95% CI: 1.28 to 1.65). Individual effect sizes ranged from 0.90 (Jolly, 2012 [43]) to 8.27 (Froozani, 1999 [29]). Substantial heterogeneity was observed (*I*^2^ = 80.2%) (Figure 2). The funnel plot assessing publication bias showed an asymmetrical distribution of studies, which suggests the potential presence of publication bias (Figure S1). When examining the correlation between control group baseline rates and the observed effect sizes, a negative correlation was found (r = −0.32; *p* = 0.10), indicating a potential trend toward larger effects in settings with lower baseline rates.

Sensitivity analyses were conducted for the (i) dose response of the BFHI steps, (ii) study design, (iii) countries’ economic status (classified by the World Bank gross national income), and (iv) follow-up periods.

### 3.7. Sensitivity Analysis for the Dose–Response Effect of BFHI Steps on EBF

**Figure 3:**
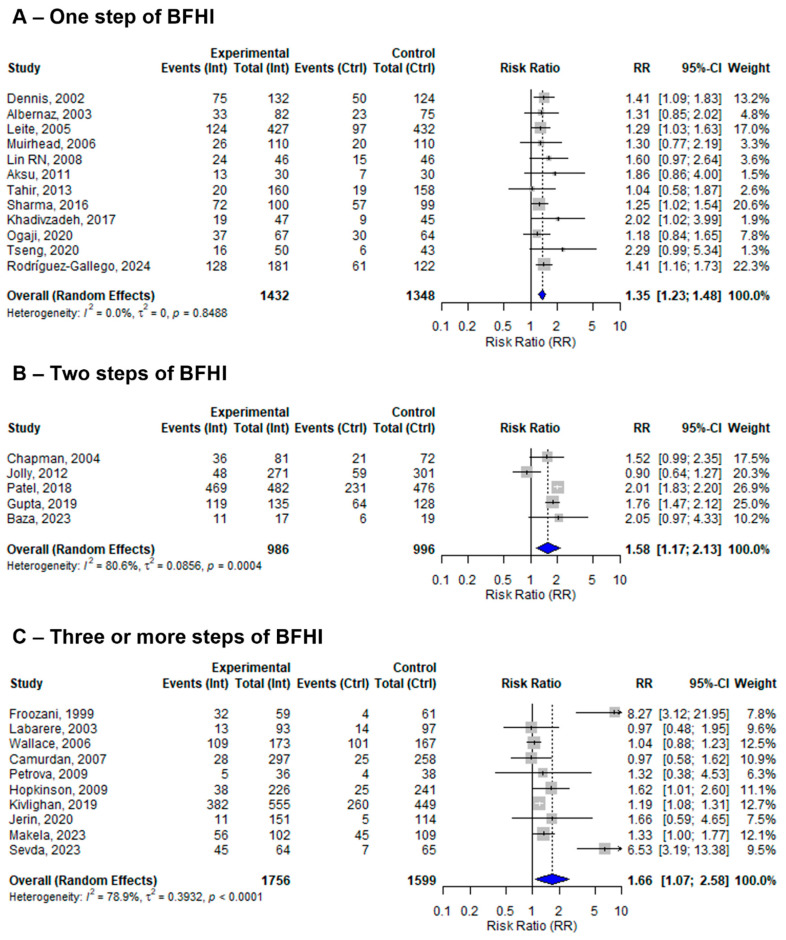
A random-effects meta-analysis comparing the risk ratio of EBF among mother–infant pairs that received (**A**) only one step, (**B**) two steps, or (**C**) three or more steps of BFHI intervention with those who received only routine care. The blue diamond is the pooled estimate. The arrow indicates estimates greater than 10 (as the x-axis ends at 10) [16,17,18,19,20,21,29,30,31,32,33,34,35,36,38,39,40,41,42,43,44,45,46,47,48,50,51].

Among the 27 included studies, 12 assessed only one step, 6 assessed two steps, and the remaining 9 assessed three or more steps of the BFHI. The pooled effect sizes of one, two, and three or more steps of BFHI interventions compared to routine care were 1.34 (95% CI: 1.22 to 1.47; *I*^2^ = 0.0%), 1.41 (95%; CI: 1.09 to 1.81; *I*^2^ = 92.7%), and 1.88 (95% CI: 1.15 to 3.07; *I*^2^ = 84.9%), respectively (Figure 3).

### 3.8. Sensitivity Analyses by Study Design

**Figure 4:**
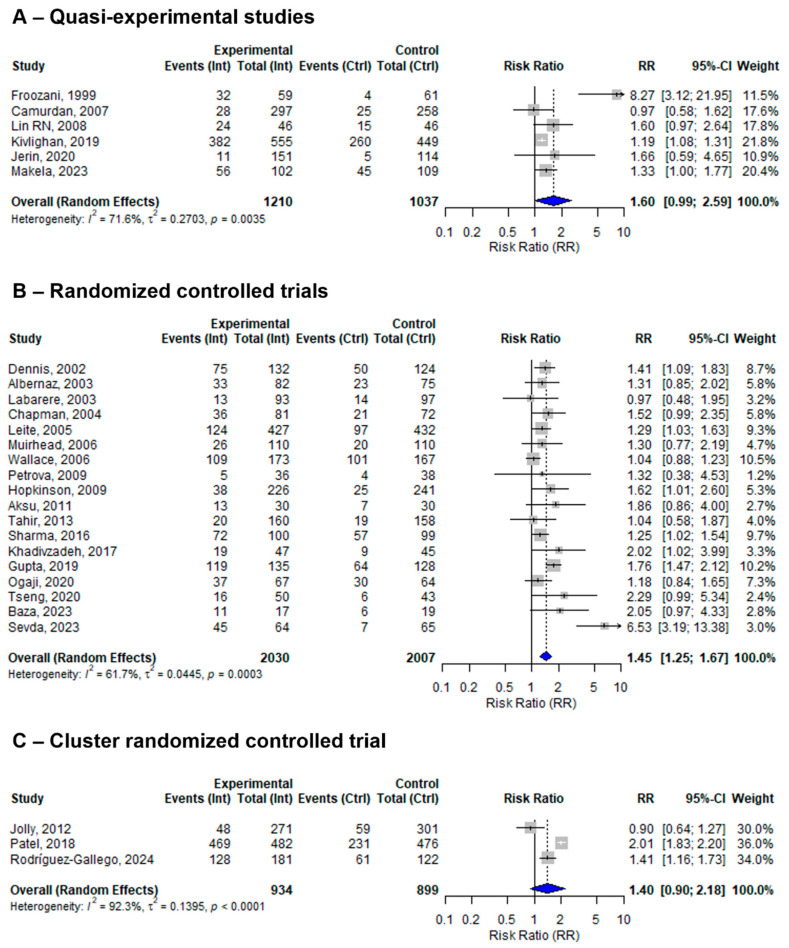
A random-effects meta-analysis comparing the risk ratio of EBF among mother–infant pairs that received BFHI intervention with those who received only routine care for (**A**) quasi-experimental studies, (**B**) RCTs, and (**C**) cluster RCTs. The blue diamond is the pooled estimate. The arrow indicates estimates greater than 10 (as the x-axis ends at 10) [16,17,18,19,20,21,29,30,31,32,33,34,35,36,38,39,40,41,42,43,44,45,46,47,48,50,51].

The 27 included studies assessed the effect of at least one BFHI step on EBF using different study designs. Five studies were quasi-experimental, nineteen were individual RCTs, and the remaining three were cluster RCTs. The pooled effect sizes of BFHI interventions compared to routine care in the quasi-experimental, individual RCT, and cluster RCT studies were 1.73 (95% CI: 1.10 to 2.72; *I*^2^ = 71%), 1.36 (95% CI: 1.20 to 1.53; *I*^2^ = 53.4%), and 1.40 (95% CI: 0.90 to 2.18; *I*^2^ = 92.3%), respectively (Figure 4).

### 3.9. Sensitivity Analysis by Countries’ Economic Status

**Figure 5:**
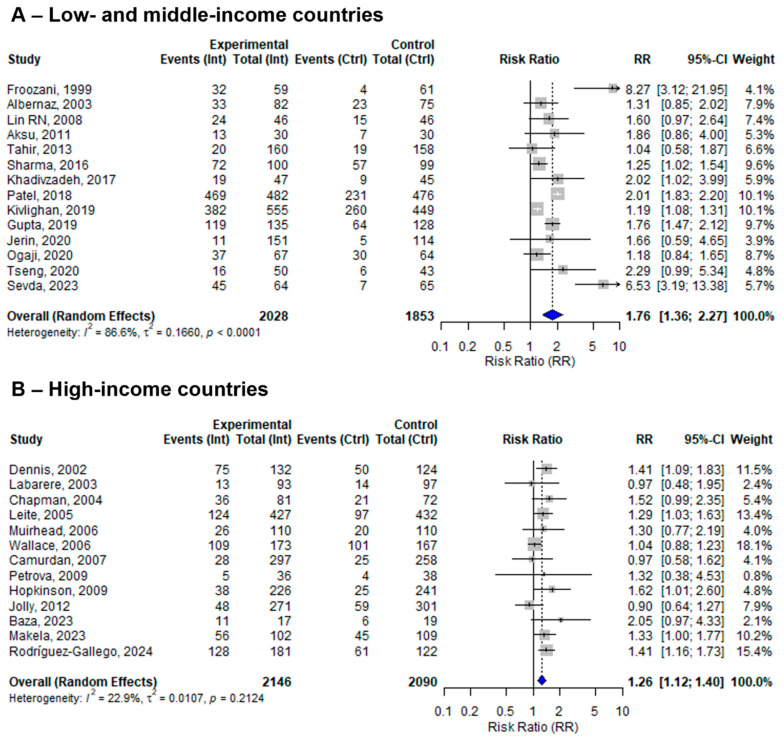
A random-effects meta-analysis comparing the risk ratio of EBF among mother–infant pairs that received BFHI intervention with those who received only routine care in (**A**) low- and middle-income countries and (**B**) high-income countries. The blue diamond is the pooled estimate. The arrow indicates estimates greater than 10 (as the x-axis ends at 10) [16,17,18,19,20,21,29,30,32,33,34,35,36,38,39,40,41,42,43,44,45,46,47,48,50,51].

Among the 27 included studies, 14 were conducted in low- and middle-income countries (LMICs), while 13 were conducted in high-income countries. The pooled effect sizes of BFHI interventions compared to routine care were 1.76 (95% CI: 1.36 to 2.27; *I*^2^ = 86.6%) in LMICs and 1.26 (95% CI: 1.12 to 1.40; *I*^2^ = 22.9%) in high-income countries (Figure 5).

### 3.10. Sensitivity Analysis by Follow-Up Period

**Figure 6:**
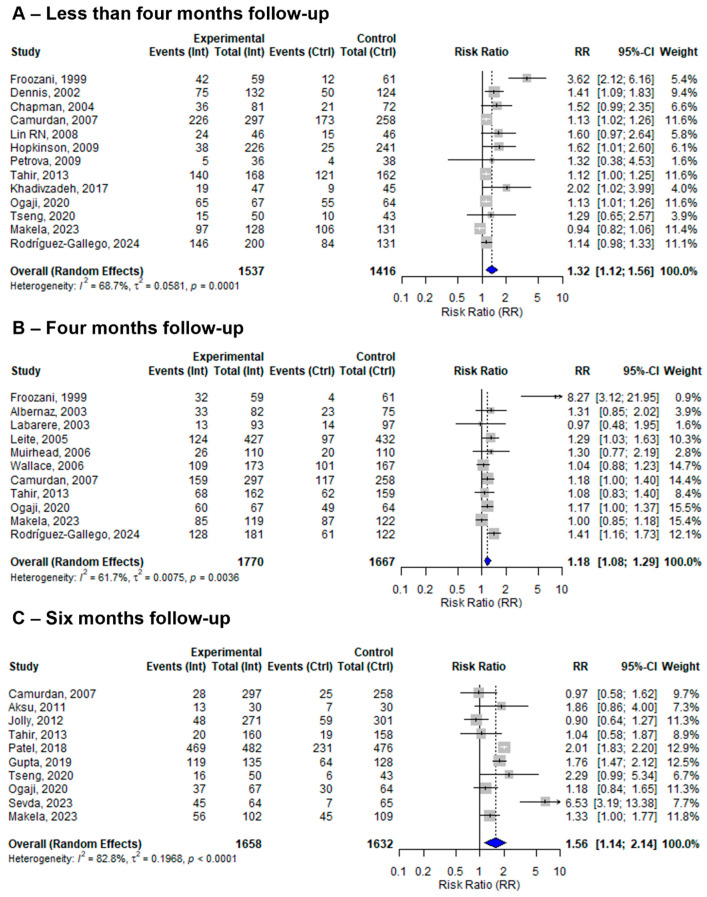
A random-effects meta-analysis comparing the risk ratio of (**A**) less than four months, (**B**) four months, and (**C**) six months of EBF among mother–infant pairs that received BFHI intervention with those that received only routine care. The blue diamond is the pooled estimate. The arrow indicates estimates greater than 10 (as the x-axis ends at 10) [16,19,20,21,29,30,31,32,33,34,35,36,38,39,40,41,42,43,44,46,47,48,50].

Among the twenty-seven included studies, four evaluated EBF at four and six months, seven reported EBF only at four months, six reported EBF only at six months, and the remaining ten studies reported EBF within less than four months of follow-up. At four months of follow-up, the pooled effect size of the BFHI intervention compared to routine care was 1.18 (95% CI: 1.08 to 1.29). The individual effect sizes ranged from 0.97 (Labarere, 2003) [32] to 8.27 (Froozani, 1999) [29], with moderate heterogeneity (*I*^2^ = 61.7%) (Figure 6). On the ther hand, from the 10 studies assessing EBF at six months, the pooled effect size of BFHI interventions compared to routine care was 1.56 (95% CI: 1.14 to 2.14). Individual effect sizes ranged from 0.90 (Jolly, 2012) [43] to 6.53 (Sevda, 2023) [19], and substantial heterogeneity among studies was observed (*I*^2^ = 82.8%) (Figure 6).

To explore the role of countries’ economic status on other sensitivity analyses, additional subgroup analyses revealed that effect sizes were consistently larger in studies conducted in LMICs compared to HICs. Furthermore, studies from LMICs showed increased heterogeneity compared to studies from HICs (Supplementary Figures S3–S5).

### 3.11. BFHI and Early Initiation of Breastfeeding

**Figure 7:**
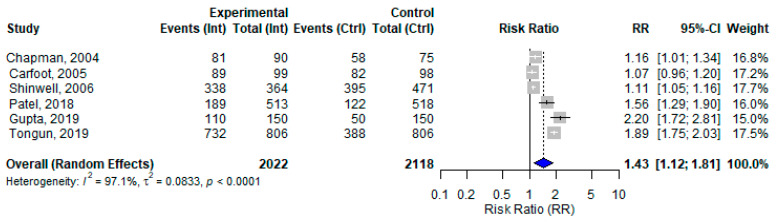
A random-effects meta-analysis comparing the risk ratio of EIBF among mother–infant pairs that received the BFHI intervention and those that received only routine care. The blue diamond is the pooled estimate [27,33,37,47,48,49].

Six studies assessed the effect of BFHI interventions on EIBF. The pooled effect size was 1.43 (95% CI: 1.12 to 1.81). The individual effect sizes ranged from 1.07 (Carfoot, 2005) [27] to 2.20 (Gupta, 2019) [48], with considerable heterogeneity across studies (*I*^2^ = 97.1%) (Figure 7). The funnel plot assessing publication bias was relatively symmetrical, which suggests a low likelihood of publication bias (Figure S2). When examining the correlation between control group baseline rates and the observed effect sizes, a strong negative correlation was found (r = −0.83; *p* = 0.04), suggesting that BFHI interventions tended to have a greater impact in settings with lower baseline initiation rates. However, this finding is based on a small number of studies and should therefore be interpreted with caution.

### 3.12. Level of Evidence

The overall level of evidence for the effectiveness of BFHI interventions on EIBF and EBF was evaluated as low to moderate (Table 3). This is due to methodological limitations, including the risk of bias in several studies and variations in the BFHI steps implemented.

## 4. Discussion

This systematic review and meta-analysis evaluated the effectiveness of the BFHI on EIBF and EBF. A total of thirty studies were included, of which twenty-four reported only EBF in different follow-up periods, three reported only EIBF, and the remaining three assessed both.

Overall, the BFHI intervention demonstrated a significant positive effect on exclusive breastfeeding practices up to six months. Most of the included studies focused on one or two specific BFHI steps. For instance, the study of Gupta et al. [48] showed a modest improvement in the early initiation of breastfeeding, possibly due to the prenatal initiation of the intervention, which was delivered repeatedly with demonstrations during antenatal care, potentially raising awareness and positively influencing maternal attitudes. Similarly, Froozani et al. [29] reported significant improvement in exclusive breastfeeding at four months, and Sevda et al. [19] found a marked increase in exclusive breastfeeding at six months. Both studies emphasized the importance of continuous breastfeeding education and support, suggesting that such interventions may lead to positive behavioral changes among mothers.

Overall, there is considerable heterogeneity among the studies included in this review. This heterogeneity may arise from differences in the number of steps implemented, which step is implemented, the study design, the sample size of the study, the follow-up period, the quality of the study, and the context. Additionally, the studies often focused on one or a few specific steps of the BFHI, which could have contributed to the observed variability. Furthermore, due to the inconsistent reporting of accreditation status across studies, it was not possible to distinguish between full and partial implementation of the BFHI. This could also be a potential source of heterogeneity.

The estimates increased based on the number of BFHI steps implemented. The sensitivity analysis showed a progressive increment in the estimates. Studies that investigated at least three or more steps of the BFHI showed stronger estimates compared to those that assessed two steps, and studies that implemented only one step showed a lesser but significant effect on EBF. Therefore, the implementation of multi-step BFHI intervention is more impactful and promising in terms of improving breastfeeding outcomes. However, it shows considerable heterogeneity, which might be due to contextual or design effects. So, the adaptation and fidelity of the intervention are very important to consider based on the context.

The sensitivity analysis revealed moderate variation within individual RCTs, which could be attributed to the predominance of RCTs among the included studies, compared to quasi-experimental studies and cluster RCTs. While RCTs are considered the gold standard for evaluating interventions and are designed to control for confounding factors through randomization [52], they still exhibit moderate variation, which could be due to differences in sample size, baseline characteristics, and the delivery of interventions. In contrast, quasi-experimental studies and cluster RCTs tend to show greater heterogeneity, which could be due to non-random assignment, variations in cluster-level interventions, and contextual factors that influence outcomes differently across study settings. Given the positive effect of the intervention, further studies are needed to evaluate the implementation of the full ten-step BFHI guidelines. Future research should focus on generating more robust evidence through high-quality, well-designed RCTs.

Notably, the effect of the BFHI intervention was significant and consistent in HICs. This may be attributed to better-equipped maternity facilities, more comprehensive and continuous breastfeeding training for staff, stronger monitoring and implementation of the BFHI, and a homogeneous healthcare system. However, this review demonstrated high heterogeneity in LMICs. This might be due to the different tailoring of interventions, local infrastructure, or socio-economic and cultural differences, which probably affect the appropriate implementation of the intervention. Additionally, studies only focused on one or a few steps of the BFHI intervention. Our subgroup analysis further confirmed that effect sizes were consistently larger in LMICs compared to high-income countries, although the variability was also considerably higher in these settings. Given the limited number of studies and the substantial heterogeneity in LMICs, these findings should be interpreted with caution. Therefore, in LMICs, there should be increasing research efforts to ensure the well-designed implementation of the BFHI intervention, and contextual adaptation of its fidelity is crucial for enabling further reproducibility of the method and intervention.

The findings of the sensitivity analysis by follow-up period demonstrated improvements in EBF at less than four months (13 studies), at four months (11 studies), and at six months (10 studies) from a total of 27 included studies compared to routine care. Some of the studies repeatedly assessed EBF at different follow-up periods. These findings align with previously published systematic reviews [53,54], although those reviews investigated the different steps of the BFHI and yielded inconclusive evidence.

This review assessed the effect of the BFHI on EIBF from six included studies reporting the initiation of breastfeeding within the first hour of birth. Despite the variability and limitations in the individual studies, the pooled estimate indicated a statistically significant impact of the BFHI on EIBF and EBF. The observed heterogeneity might be due to differences in the BFHI steps implemented, study design, sample size, randomization methods, or population characteristics. Our findings align with previous meta-analyses, which also reported that specific BFHI steps, such as step 3, were associated with improved EIBF [55].

While the heterogeneity observed across studies requires careful interpretation, the overall findings of this review are consistent with and support the WHO recommendations [9], which promote EIBF and EBF. The observed positive outcome in this review is the effect of at least one or more steps of the BFHI intervention, underscoring the program’s value. The WHO and UNICEF designed the BFHI to improve the status of early initiation and exclusive breastfeeding practices and help mothers and infants benefit from the full advantages of breastfeeding [56]. Policymakers must prioritize the implementation of this BFHI program in maternity healthcare facilities, which can contribute towards achieving Sustainable Development Goal 3, Target 2, which aims to end preventable deaths of newborns and deaths of children under five.

### 4.1. Clinical Implications

This meta-analysis highlights that the BFHI intervention, especially its multi-step implementation, can improve breastfeeding outcomes. Facilities implementing the BFHI steps are more likely to enhance breastfeeding practices compared to those with routine care. Additionally, this review’s findings have the potential to encourage facilities to implement at least one step of the BFHI, since increasing the number of steps showed stronger estimates. However, the current evidence remains limited, and efforts to strengthen the feasibility and consistency of BFHI implementation are essential to improve breastfeeding outcomes.

### 4.2. Strengths and Limitations of This Review

This systematic review and meta-analysis was conducted following rigorous methodological standards. A comprehensive search was performed across four major databases without restrictions on the time or setting. The review also followed a more inclusive approach by considering studies cited in the initially identified papers. Additionally, this review includes studies that implemented at least one step of the BFHI.

Nonetheless, several limitations should be acknowledged. The review included English-language papers, which may have excluded relevant non-English publications. Only six studies reported EIBF, while the follow-up periods for EBF varied across studies. Additionally, most studies were conducted in high- and middle-income countries, limiting the generalizability of the findings to low-resource settings. Few studies reported on infant health outcomes in relation to breastfeeding, which would be valuable for assessing the broader impact of BFHI interventions. It was not possible to distinguish between full (i.e., with accreditation) and partial implementation of the BFHI, as this information was not available in the included studies. Furthermore, it would be interesting to investigate if certain steps are more effective in incerasing EIBF and EBF. However, there was insufficient data available on single steps, as most studies implemented multiple steps of the BFHI.

## 5. Conclusions

This systematic review and meta-analysis showed that BFHI interventions positively affect EIBF and EBF, particularly their multi-step implementation. Our findings suggest that the effectiveness of the BFHI on EBF is greater in studies implementing multiple or comprehensive steps, indicating that broader adoption of the initiative may yield stronger effects than isolated or partial implementations. Despite limitations in the available data and heterogeneity between the studies, health facilities should be encouraged to implement BFHI strategies and strengthen healthcare providers’ skills through training. Continuous monitoring and evaluation are vital to optimizing breastfeeding outcomes. Future research should focus on well-designed, large-scale randomized controlled trials to provide stronger evidence on the effectiveness of full BFHI implementation, especially in low-income countries.

## Figures and Tables

**Table 1:** Characteristics of included studies.

Author, Year	Country	Study-Design	Sample Size	BFHI Steps	Duration of Follow-Up	Outcome
Froozani, 1999 [29]	Iran	Quasi-experimental	120	Steps 2, 5, 6, 8, and 10	4 MO	EBF
Dennis, 2002 [30]	Toronto	Individual RCT	256	Step 10	3 MO	EBF
Albernaz, 2003 [31]	Brazil	Individual RCT	188	Step 10	4 MO	EBF
Labarere, 2003 [32]	France	Individual RCT	210	Steps 2, 5, and 10	4 MO	EBF
Chapman, 2004 [33]	USA	Individual RCT	165	Steps 3 and 10	3 MO	EBF and EIBF
Leite, 2005 [34]	USA	Individual RCT	1003	Step 10	4 MO	EBF
Carfoot, 2005 [27]	UK	Individual RCT	204	Step 4	1 h	EIBF
Muirhead, 2006 [35]	UK	Individual RCT	220	Step 10	4 MO	EBF
Wallace, 2006 [36]	UK	Individual RCT	370	Steps 2, 3, 5, and 8	4 MO	EBF
Shinwell, 2006 [37]	Israel	Quasi-experimental	835	Step 2	1 h	EIBF
Çamurdan, 2007 [38]	Greece	Quasi-experimental	555	All steps	6 MO	EBF
Lin RN, 2008 [39]	China	Quasi-experimental	92	Step 3	1 MO	EBF
Hopkinson, 2009 [40]	USA	Individual RCT	104	Steps 2, 5, 8, and 10	3 MO	EBF
Petrova, 2009 [41]	USA	Individual RCT	467	Steps 3, 5, 6, 8, and 10	1 MO	EBF
Aksu, 2011 [42]	Turkey	Individual RCT	60	Step 10	6 MO	EBF
Jolly, 2012 [43]	UK	Cluster RCT	2724	Steps 3 and 10	6 MO	EBF
Tahir, 2013 [44]	Malaysia	Individual RCT	357	Step 10	6 MO	EBF
Sharma, 2016 [45]	India	Individual RCT	200	Step 4	6 WK	EBF
Khadivzadeh, 2017 [46]	Iran	Individual RCT	92	Step 4	1 MO	EBF
Patel, 2018 [47]	India	Cluster RCT	1031	Steps 3 and 10	6 MO	EBF and EIBF
Kivlighan, 2019 [17]	Mexico	Quasi-experimental	1004	Steps 4–9	6 WK	EBF
Gupta, 2019 [48]	India	Individual RCT	300	Steps 3 and 10	6 MO	EBF and EIBF
Tongun, 2019 [49]	Sudan	Quasi-experimental	1612	Step 2	1 h	EIBF
Jerin, 2020 [18]	Bangladesh	Quasi-experimental	265	Steps 2, 3, 4, 5, 8, and 10	5 MO	EBF
Ogaji, 2020 [21]	Nigeria	Individual RCT	150	Step 10	6 MO	EBF
Tseng, 2020 [50]	China	Individual RCT	104	Step 3	6 MO	EBF
Baza, 2023 [51]	USA	Individual RCT	40	Steps 5 and 10	6 WK	EBF
Makela, 2023 [20]	Finland	Quasi-experimental	325	All steps	6 MO	EBF
Sevda, 2023 [19]	Turkey	Individual RCT	128	Steps 5, 9, and 10	6 MO	EBF
Rodríguez-Gallego, 2024 [16]	Spain	Cluster RCT	382	Step 10	4 MO	EBF

MO = month; WK = week; EBF = exclusive breastfeeding; EIBF = early initiation of breastfeeding.

**Table 2:** Risk-of-bias assessment for the 30 included studies. Bias was categorized as high (red), some (yellow), or low (green). D1: Bias arising from the randomization process; D2: Bias due to deviation from the intended intervention; D3: Bias due to missing outcome data; D4: Bias in the measurement of the outcome; D5: Bias in the selection of the reported result.

Author	Risk-of-Bias Domain					
	D1	D2	D3	D4	D5	Overall
Froozani, 1999 [29]						
Dennis, 2002 [30]						
Albernaz, 2003 [31]						
Labarere, 2003 [32]						
Chapman, 2004 [33]						
Leite, 2005 [34]						
Carfoot, 2005 [27]						
Muirhead, 2006 [35]						
Wallace, 2006 [36]						
Shinwell, 2006 [37]						
Çamurdan, 2007 [38]						
Lin RN, 2008 [39]						
Petrova, 2009 [41]						
Hopkinson, 2009 [40]						
Aksu, 2011 [42]						
Jolly, 2012 [43]						
Tahir, 2013 [44]						
Sharma, 2016 [45]						
Khadivzadeh, 2017 [46]						
Patel, 2018 [47]						
Kivlighan, 2019 [17]						
Gupta, 2019 [48]						
Tongun, 2019 [49]						
Jerin, 2020 [18]						
Ogaji, 2020 [21]						
Tseng, 2020 [50]						
Baza, 2023 [51]						
Makela, 2023 [20]						
Sevda, 2023 [19]						
Rodríguez-Gallego, 2024 [16]						

**Table 3:** Quality of evidence, appraised using the Grading of Recommendations, Assessments, Development, and Evaluation (GRADE) method.

Outcome		Studies	Risk of Bias	Inconsistency	Indirectness	Imprecision	Publication Bias	Quality of the Evidence
EBF: Main analysis		27	Serious	Serious	Not serious	Not serious	Serious	Low
EBF: Sensitivity by study design	Quasi-experimental	5	Very Serious	Very Serious	Not serious	Serious	Serious	Low
	Individual RCT	19	Serious	Serious	Not serious	Not serious	Not serious	Medium
	Cluster RCT	3	Serious	Very Serious	Not Serious	Serious	Serious	Low
EBF: Sensitivity by economic status	LMICS	14	Very Serious	Serious	Not serious	Not serious	Serious	Low
	High	13	Serious	Not serious	Not serious	Not serious	Serious	Medium
EBF: Sensitivity by follow-up period	EBF 4 months	11	Serious	Serious	Not serious	Not serious	Serious	Medium
	EBF 6 months	10	Very serious	Very Serious	Not serious	Not serious	Serious	Low
EIBF: Main analysis		6	Very Serious	Very Serious	Not serious	Not serious	Serious	Low

EIBF: early initiation of breastfeeding; EBF: exclusive breastfeeding.

## Data Availability

The raw data supporting the conclusions of this article will be made available by the authors on request.

## Supplementary Materials

The following supporting information can be downloaded at: https://www.mdpi.com/article/10.3390/nu17142283/s1, Figure S1: Funnel plot of exclusive breastfeeding practice at different follow-up periods among BFHI-received versus routine care; Figure S2: Funnel plot of early initiation of breastfeeding practice among BFHI-received versus routine care; Figure S3: A random effect meta-analysis comparing the risk ratio of EBF among mother-infant pairs that received (A) only one step of BFHI in LMIC, (B) only one step of BFHI in HIC, (C) two steps of BFHI in LMIC, (D) two steps of BFHI in HIC, (E) three or more steps of BFHI in LMIC, and (F) three or more steps of BFHI in HIC; Figure S4: A random effect meta-analysis comparing the risk ratio of EBF among mother-infant pairs that received BFHI intervention with receiving only routine care for (A) RCTs in LMICs, (B) RCTs in HICs, (C) quasi-experimental studies in LMICs, and (D) quasi-experimental studies in HICs. Due to the limited number of studies for cluster RCTs, no subgroup analysis for countries’ economic class was performed; Figure S5: A random effect meta-analysis comparing the risk ratio of (A) less than four months of EBF in LMICs, (B) less than four months of EBF in HICs, (C) four months of EBF in LMICs, (D) four months of EBF in HICs, (E) six months of EBF in LMICs, and (F) six months of EBF in HICs among mother-infant pairs that received BFHI intervention with those that received only routine care.
