# Supplementary material for: Effectiveness of Baby-Friendly Hospital Initiative on Early Initiation and Exclusive Breastfeeding Practice: Systematic Review and Meta-Analysis
Source: Nutrients. 2025 Jul 10;17(14):2283. doi: 10.3390/nu17142283 (PMC12300584; doi:10.3390/nu17142283)
Supplement: Supplementary file 1 [file nutrients-17-02283-s001.zip › nutrients-3693952-supplementary.pdf]

Supplemental information

Search strategy

PUBMED Search strategy

Search: ((((((((((Neonate[Title/Abstract]) OR (Newborn[Title/Abstract])) OR (Infant[Title/Abstract])) OR ("0-6 month"[Title/Abstract])) OR (six month[Title/Abstract])) OR (("Infant, Newborn"[MeSH Terms]) OR (infant[MeSH Terms]))) OR (((((("6 month"[Title/Abstract]) OR ("5 month"[Title/Abstract])) OR ("4 month"[Title/Abstract])) OR ("3 month"[Title/Abstract])) OR ("2 month"[Title/Abstract])) OR ("1 month"[Title/Abstract]))) OR (((((("below six-month"[Title/Abstract]) OR ("Under six month"[Title/Abstract])) OR ("Nursing mothers"[Title/Abstract])) OR ("Breastfeeding mothers"[Title/Abstract])) OR ("Breastfeeding women"[Title/Abstract])) OR ("Lactating women"[Title/Abstract]))) OR ((("Lactating mother"[Title/Abstract]) OR ("Women of child bearing age"[Title/Abstract])) OR ("Women of reproductive age"[Title/Abstract]))) AND (((((((((("baby friendly hospital initiative"[Title/Abstract]) OR ("baby friendly initiative"[Title/Abstract])) OR ("friendly hospital"[Title/Abstract])) OR ("baby friendly"[Title/Abstract])) OR ("ten steps"[Title/Abstract])) OR ("10 steps"[Title/Abstract])) OR ((((((("Breastfeeding support"[Title/Abstract]) OR ("Breastfeeding education"[Title/Abstract])) OR ("Baby Friendly Community Initiative"[Title/Abstract])) OR (BFCI[Title/Abstract])) OR ("Baby-friendly"[Title/Abstract])) OR (BFI[Title/Abstract])) OR (BFHI[Title/Abstract]))) OR ((("lactation support"[Title/Abstract]) OR ("lactation consultation"[Title/Abstract])) OR ("Breastfeeding counselling"[Title/Abstract]))) OR ((((((((((((Policy[Title/Abstract]) OR (Guideline[Title/Abstract])) OR (Training[Title/Abstract])) OR (Counsel*[Title/Abstract])) OR ("ANC care"[Title/Abstract])) OR ("Skin to skin"[Title/Abstract])) OR (Support[Title/Abstract])) OR ("Exclusive feeding"[Title/Abstract])) OR ("Step 6"[Title/Abstract])) OR (Rooming-in[Title/Abstract])) OR (Rooming[Title/Abstract])) OR ("Feeding on demand"[Title/Abstract]))) OR ((((("Step 8"[Title/Abstract]) OR (Pacifier[Title/Abstract])) OR ("Community support"[Title/Abstract])) OR ("PNC care"[Title/Abstract])) OR ("peer support"[Title/Abstract])))) AND (((("Breast Feeding"[MeSH Terms]) OR (((("Breast Fed"[Title/Abstract]) OR ("Breast Feeding, Exclusive"[Title/Abstract])) OR (Breastfed[Title/Abstract])) OR (Breastfeeding[Title/Abstract]))) OR ((((("Breastfeeding, Exclusive"[Title/Abstract]) OR (Lactation[Title/Abstract])) OR ("Breastfeeding experience"[Title/Abstract])) OR (EBF[Title/Abstract])) OR (EIB[Title/Abstract]))) OR ((("Early initiation"[Title/Abstract]) OR

("Timely initiation"[Title/Abstract])) OR (lactation[Title/Abstract]))) Filters: Clinical Trial, Randomized Controlled Trial, English, from 1991 - 2025/04/01

Scopus Search Strategy

((TITLE-ABS-KEY ("Infant" OR "New-born" OR "Neonate") OR TITLE-ABS-KEY ("Under six" OR "Below six or 0-6 month" OR "under 6 month") OR TITLE-ABS-KEY ("Breastfeeding mothers" OR "Breastfeeding women") OR TITLE-ABS-KEY ("Lactating women" OR "Nursing mothers" OR "Lactating mother"))) AND ((TITLE-ABS-KEY ("Breastfeeding experience" OR ebf) OR TITLE-ABS-KEY ("Breast Feeding" OR "Breast Fed") OR TITLE-ABS-KEY ("Breast Feeding, Exclusive" OR breastfed) OR TITLE-ABS-KEY (breastfeeding OR "Breastfeeding, Exclusive") OR TITLE-ABS-KEY ("early initiation" OR "timely initiation" OR "EIB"))) AND ((TITLE-ABS-KEY (bfi OR bfhi) OR TITLE-ABS-KEY ("baby friendly hospital initiative" OR "baby friendly initiative") OR TITLE-ABS-KEY ("friendly hospital" OR "baby friendly") OR TITLE-ABS-KEY ("ten step* or " 10 steps*) OR TITLE-ABS-KEY ("Ten Steps for Healthy Feeding" OR baby-friendly)))

Web of Science

#1 (TS=("Infant" or "New-born" or "Neonate" or "Under six" or "Below six or 0-6 month" or "under 6 month" or "Breastfeeding mothers" or "Breastfeeding women" or "Lactating women" or "Nursing mothers" or "Lactating mother"))

#2 (TS=(BFI or BFHI or "baby friendly hospital initiative" or "baby friendly initiative" or "friendly hospital" or "baby friendly" or "ten step* or "10 steps* or "Ten Steps for Healthy Feeding" or Baby-friendly))

#3 (TS=("Breastfeeding experience" or EBF or "Breast Feeding" or "Breast Fed" or "Breast Feeding, Exclusive" or Breastfed or Breastfeeding or "Breastfeeding, Exclusive" or "early initiation" or "timely initiation" or "EIB"))

#4 #1 AND #2 AND #3

Ten steps of BFHI

**Step 1**	a. Comply fully with the International Code of Marketing of Breast-milk Substitutes and relevant World Health Assembly resolutions.<br>b. Have a written infant feeding policy that is routinely communicated to staff and parents.<br>c. Establish ongoing monitoring and data-management systems.
**Step 2**	Ensure that staff have sufficient knowledge, competence and skills to support breastfeeding.
**Step 3**	Discuss the importance and management of breastfeeding with pregnant women and their families.
**Step 4**	Facilitate immediate and uninterrupted skin-to-skin contact and support mothers to initiate breastfeeding as soon as possible after birth.
**Step 5**	Support mothers to initiate and maintain breastfeeding and manage common difficulties.
**Step 6**	Do not provide breastfed new-borns any food or fluids other than breast milk, unless medically indicated.
**Step 7**	Enable mothers and their infants to remain together and to practise rooming-in 24 hours a day.
**Step 8**	Support mothers to recognize and respond to their infants' cues for feeding.
**Step 9**	Counsel mothers on the use and risks of feeding bottles, teats and pacifiers.
**Step 10**	Coordinate discharge so that parents and their infants have timely access to ongoing support and care.

Supplemental Figures

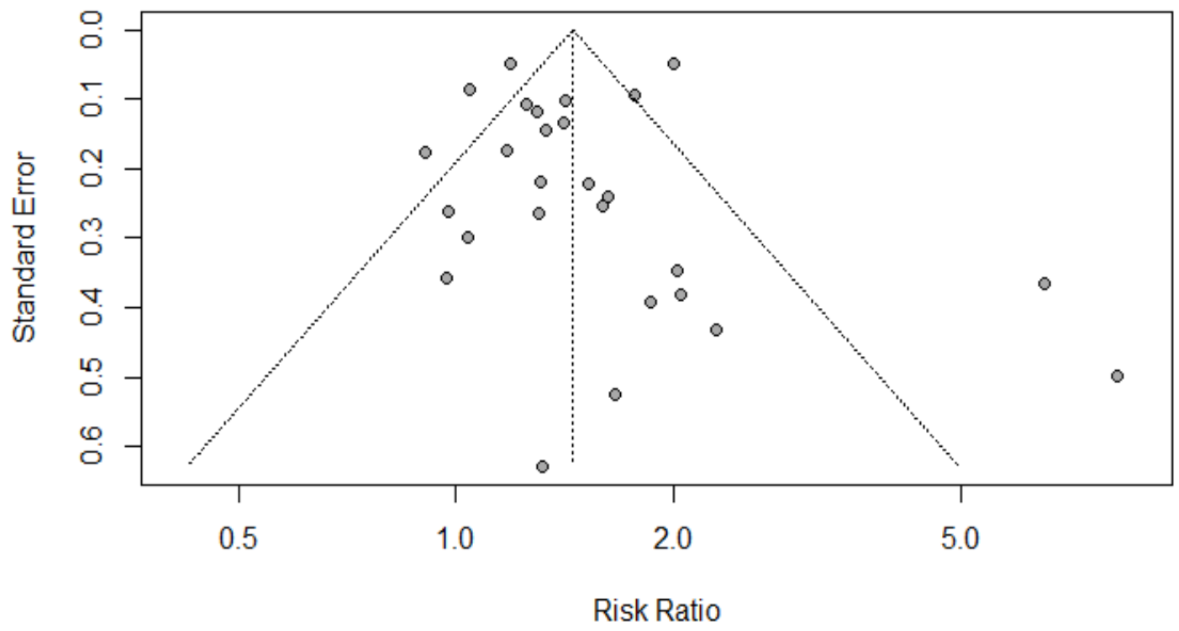

Figure S1: Funnel plot of exclusive breastfeeding practice at different follow-up periods among BFHI-received versus routine care.

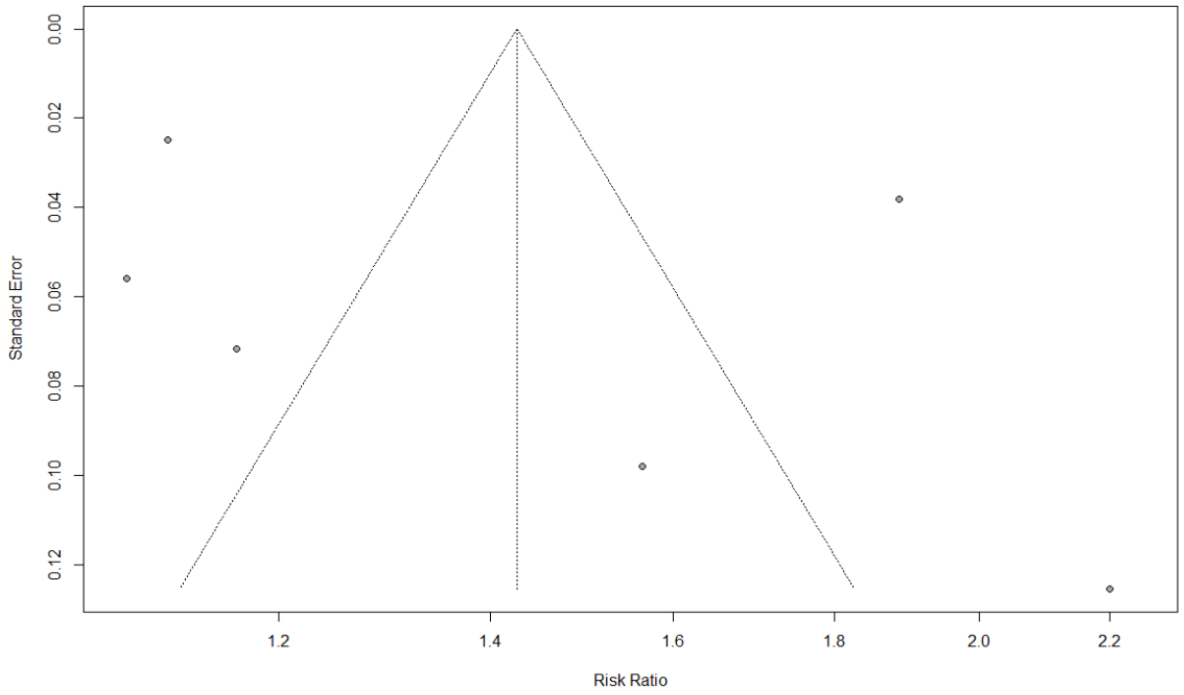

Figure S2: Funnel plot of early initiation of breastfeeding practice among BFHI-received versus routine care.

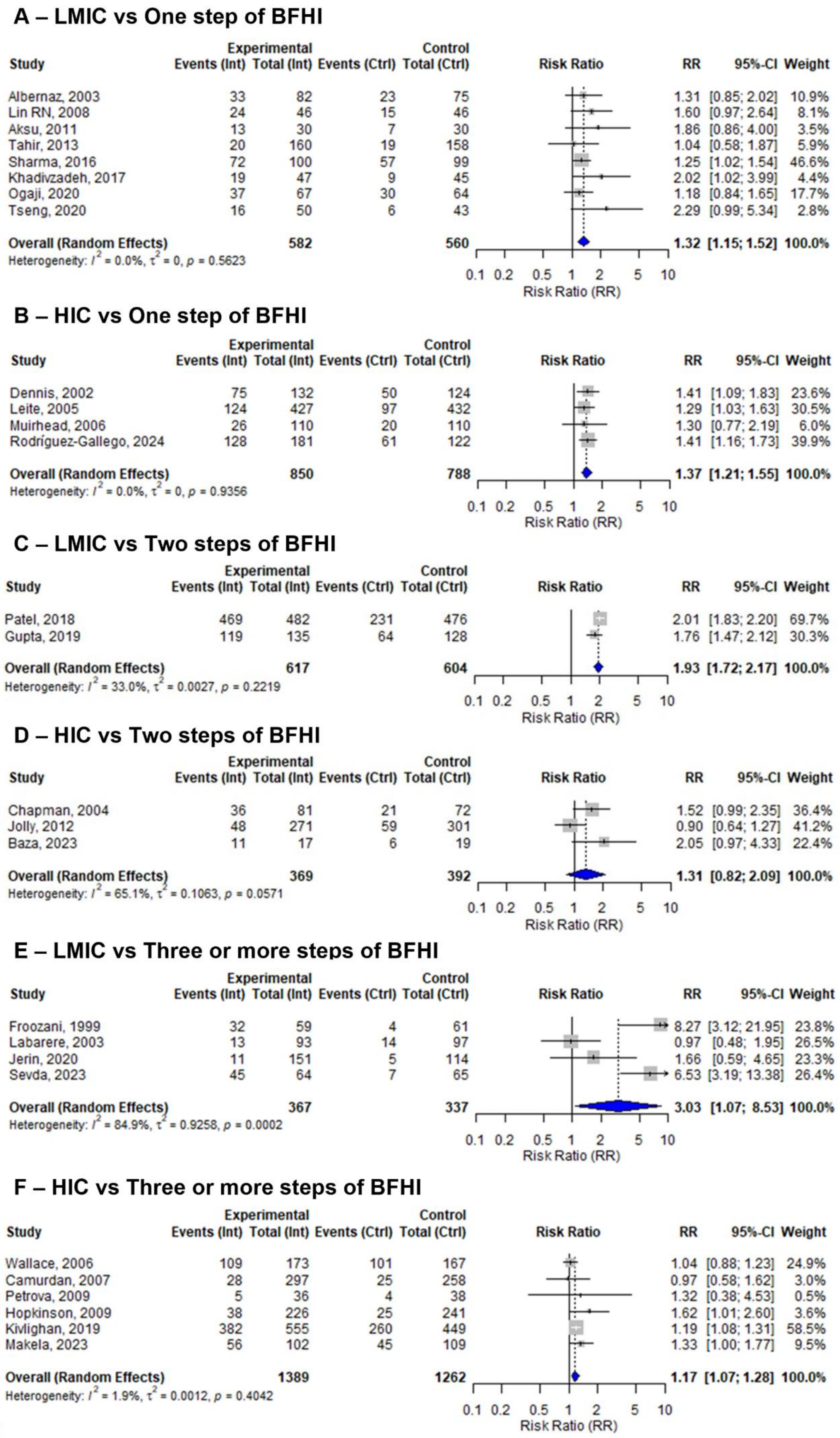
A – LMIC vs One step of BFHI
Study | Experimental Events (Int) | Total (Int) | Control Events (Ctrl) | Total (Ctrl) | Risk Ratio | RR | 95%-CI | Weight
Albernaz, 2003 33 82 23 75 1.31 [0.85; 2.02] 10.9%
Lin RN, 2008 24 46 15 46 1.60 [0.97; 2.64] 8.1%
Aksu, 2011 13 30 7 30 1.86 [0.86; 4.00] 3.5%
Tahir, 2013 20 160 19 158 1.04 [0.58; 1.87] 5.9%
Sharma, 2016 72 100 57 99 1.25 [1.02; 1.54] 46.6%
Khadivzadeh, 2017 19 47 9 45 2.02 [1.02; 3.99] 4.4%
Ogaji, 2020 37 67 30 64 1.18 [0.84; 1.65] 17.7%
Tseng, 2020 16 50 6 43 2.29 [0.99; 5.34] 2.8%
Overall (Random Effects) 582 560 1.32 [1.15; 1.52] 100.0%
Heterogeneity: I^2 = 0.0%, τ^2 = 0, p = 0.5623
0.1 0.2 0.5 1 2 5 10
Risk Ratio (RR)
B – HIC vs One step of BFHI
Dennis, 2002 75 132 50 124 1.41 [1.09; 1.83] 23.6%
Leite, 2005 124 427 97 432 1.29 [1.03; 1.63] 30.5%
Muirhead, 2006 26 110 20 110 1.30 [0.77; 2.19] 6.0%
Rodríguez-Gallego, 2024 128 181 61 122 1.41 [1.16; 1.73] 39.9%
Overall (Random Effects) 850 788 1.37 [1.21; 1.55] 100.0%
Heterogeneity: I^2 = 0.0%, τ^2 = 0, p = 0.9356
C – LMIC vs Two steps of BFHI
Patel, 2018 469 482 231 476 2.01 [1.83; 2.20] 69.7%
Gupta, 2019 119 135 64 128 1.76 [1.47; 2.12] 30.3%
Overall (Random Effects) 617 604 1.93 [1.72; 2.17] 100.0%
Heterogeneity: I^2 = 33.0%, τ^2 = 0.0027, p = 0.2219
D – HIC vs Two steps of BFHI
Chapman, 2004 36 81 21 72 1.52 [0.99; 2.35] 36.4%
Jolly, 2012 48 271 59 301 0.90 [0.64; 1.27] 41.2%
Baza, 2023 11 17 6 19 2.05 [0.97; 4.33] 22.4%
Overall (Random Effects) 369 392 1.31 [0.82; 2.09] 100.0%
Heterogeneity: I^2 = 65.1%, τ^2 = 0.1063, p = 0.0571
E – LMIC vs Three or more steps of BFHI
Froozani, 1999 32 59 4 61 8.27 [3.12; 21.95] 23.8%
Labarere, 2003 13 93 14 97 0.97 [0.48; 1.95] 26.5%
Jerin, 2020 11 151 5 114 1.66 [0.59; 4.65] 23.3%
Sevda, 2023 45 64 7 65 6.53 [3.19; 13.38] 26.4%
Overall (Random Effects) 367 337 3.03 [1.07; 8.53] 100.0%
Heterogeneity: I^2 = 84.9%, τ^2 = 0.9258, p = 0.0002
F – HIC vs Three or more steps of BFHI
Wallace, 2006 109 173 101 167 1.04 [0.88; 1.23] 24.9%
Camurdan, 2007 28 297 25 258 0.97 [0.58; 1.62] 3.0%
Petrova, 2009 5 36 4 38 1.32 [0.38; 4.53] 0.5%
Hopkinson, 2009 38 226 25 241 1.62 [1.01; 2.60] 3.6%
Kivlighan, 2019 382 555 260 449 1.19 [1.08; 1.31] 58.5%
Makela, 2023 56 102 45 109 1.33 [1.00; 1.77] 9.5%
Overall (Random Effects) 1389 1262 1.17 [1.07; 1.28] 100.0%
Heterogeneity: I^2 = 1.9%, τ^2 = 0.0012, p = 0.4042

Figure S3: A random effect meta-analysis comparing the risk ratio of EBF among mother-infant pairs that received (A) only one step of BFHI in LMIC, (B) only one step of BFHI in HIC, (C) two steps of BFHI in LMIC, (D) two steps of BFHI in HIC, (E) three or more steps of BFHI in LMIC, and (F) three or more steps of BFHI in HIC.

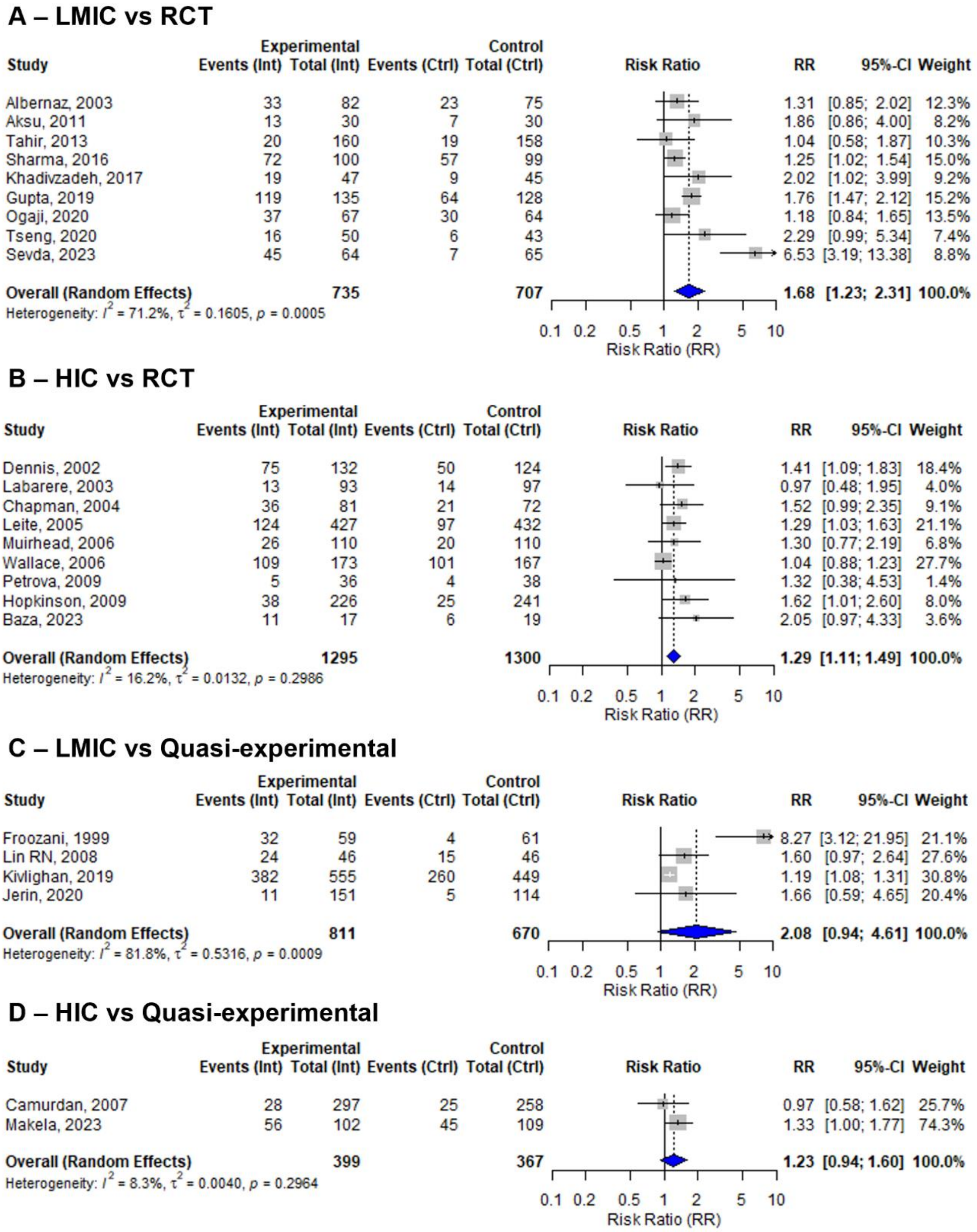

Figure S4: A random effect meta-analysis comparing the risk ratio of EBF among mother-infant pairs that received BFHI intervention with receiving only routine care for (A) RCTs in LMICs, (B) RCTs in HICs, (C) quasi-experimental studies in LMICs, and (D) quasi-experimental studies in HICs. Due to the limited number of studies for cluster RCTs, no subgroup analysis for countries' economic class was performed.

A – LMIC vs Less than 4 months follow-up

Study	Experimental Events (Int)	Experimental Total (Int)	Control Events (Ctrl)	Control Total (Ctrl)	RR	95%-CI	Weight
Froozani, 1999	42	59	12	61	3.62	[2.12; 6.16]	15.2%
Lin RN, 2008	24	46	15	46	1.60	[0.97; 2.64]	15.8%
Tahir, 2013	140	168	121	162	1.12	[1.00; 1.25]	22.0%
Khadivzadeh, 2017	19	47	9	45	2.02	[1.02; 3.99]	12.6%
Ogaji, 2020	65	67	55	64	1.13	[1.01; 1.26]	22.0%
Tseng, 2020	15	50	10	43	1.29	[0.65; 2.57]	12.4%
**Overall (Random Effects)**		**437**		**421**	**1.55**	**[1.08; 2.23]**	**100.0%**

Heterogeneity: I^2 = 77.7%, τ^2 = 0.1530, p = 0.0004

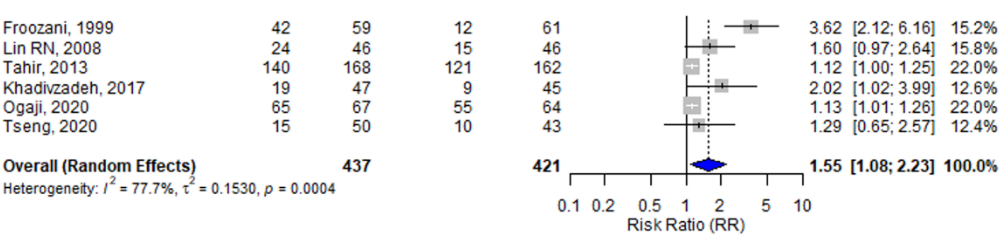

B – HIC vs Less than 4 months follow-up

Study	Experimental Events (Int)	Experimental Total (Int)	Control Events (Ctrl)	Control Total (Ctrl)	RR	95%-CI	Weight
Dennis, 2002	75	132	50	124	1.41	[1.09; 1.83]	14.9%
Chapman, 2004	36	81	21	72	1.52	[0.99; 2.35]	8.2%
Camurdan, 2007	226	297	173	258	1.13	[1.02; 1.26]	24.1%
Hopkinson, 2009	38	226	25	241	1.62	[1.01; 2.60]	7.3%
Petrova, 2009	5	36	4	38	1.32	[0.38; 4.53]	1.4%
Makela, 2023	97	128	106	131	0.94	[0.82; 1.06]	22.8%
Rodríguez-Gallego, 2024	146	200	84	131	1.14	[0.98; 1.33]	21.3%
**Overall (Random Effects)**		**1100**		**995**	**1.18**	**[1.02; 1.37]**	**100.0%**

Heterogeneity: I^2 = 59.5%, τ^2 = 0.0208, p = 0.0218

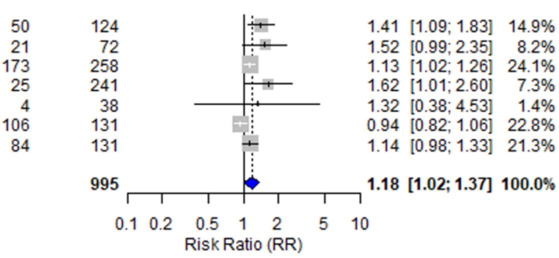

C – LMIC vs 4 months follow-up

Study	Experimental Events (Int)	Experimental Total (Int)	Control Events (Ctrl)	Control Total (Ctrl)	RR	95%-CI	Weight
Froozani, 1999	32	59	4	61	8.27	[3.12; 21.95]	19.9%
Albernaz, 2003	33	82	23	75	1.31	[0.85; 2.02]	25.7%
Tahir, 2013	68	162	62	159	1.08	[0.83; 1.40]	26.9%
Ogaji, 2020	60	67	49	64	1.17	[1.00; 1.37]	27.4%
**Overall (Random Effects)**		**370**		**359**	**1.74**	**[0.77; 3.94]**	**100.0%**

Heterogeneity: I^2 = 81.2%, τ^2 = 0.6273, p = 0.0012

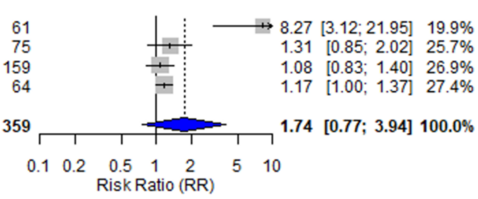

D – HIC vs 4 months follow-up

Study	Experimental Events (Int)	Experimental Total (Int)	Control Events (Ctrl)	Control Total (Ctrl)	RR	95%-CI	Weight
Labarere, 2003	13	93	14	97	0.97	[0.48; 1.95]	2.6%
Leite, 2005	124	427	97	432	1.29	[1.03; 1.63]	14.8%
Muirhead, 2006	26	110	20	110	1.30	[0.77; 2.19]	4.4%
Wallace, 2006	109	173	101	167	1.04	[0.88; 1.23]	20.2%
Camurdan, 2007	159	297	117	258	1.18	[1.00; 1.40]	19.9%
Makela, 2023	85	119	87	122	1.00	[0.85; 1.18]	21.0%
Rodríguez-Gallego, 2024	128	181	61	122	1.41	[1.16; 1.73]	17.1%
**Overall (Random Effects)**		**1400**		**1308**	**1.16**	**[1.03; 1.30]**	**100.0%**

Heterogeneity: I^2 = 38.9%, τ^2 = 0.0101, p = 0.1325

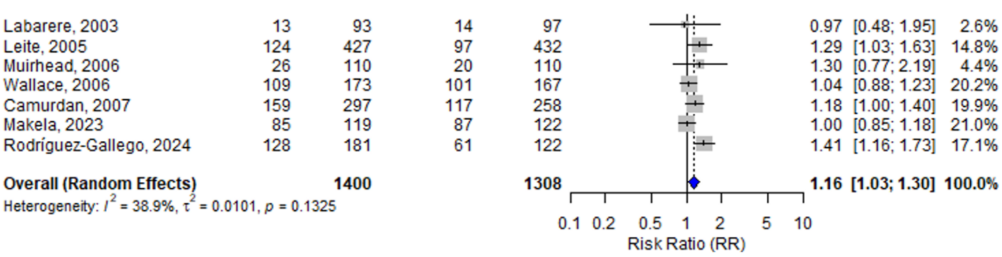

E – LMIC vs 6 months follow-up

Study	Experimental Events (Int)	Experimental Total (Int)	Control Events (Ctrl)	Control Total (Ctrl)	RR	95%-CI	Weight
Aksu, 2011	13	30	7	30	1.86	[0.86; 4.00]	11.0%
Tahir, 2013	20	160	19	158	1.04	[0.58; 1.87]	13.4%
Patel, 2018	469	482	231	476	2.01	[1.83; 2.20]	18.8%
Gupta, 2019	119	135	64	128	1.76	[1.47; 2.12]	18.3%
Tseng, 2020	16	50	6	43	2.29	[0.99; 5.34]	10.1%
Ogaji, 2020	37	67	30	64	1.18	[0.84; 1.65]	16.7%
Sevda, 2023	45	64	7	65	6.53	[3.19; 13.38]	11.7%
**Overall (Random Effects)**		**988**		**964**	**1.89**	**[1.28; 2.80]**	**100.0%**

Heterogeneity: I^2 = 76.1%, τ^2 = 0.2111, p = 0.0003

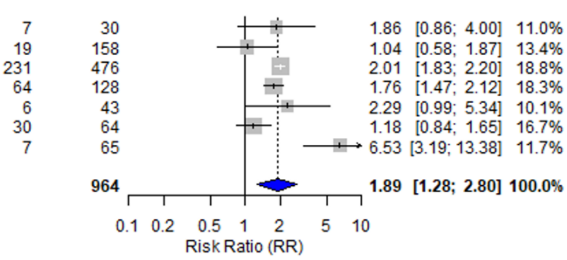

F – HIC vs 6 months follow-up

Study	Experimental Events (Int)	Experimental Total (Int)	Control Events (Ctrl)	Control Total (Ctrl)	RR	95%-CI	Weight
Camurdan, 2007	28	297	25	258	0.97	[0.58; 1.62]	21.1%
Jolly, 2012	48	271	59	301	0.90	[0.64; 1.27]	35.6%
Makela, 2023	56	102	45	109	1.33	[1.00; 1.77]	43.3%
**Overall (Random Effects)**		**670**		**668**	**1.08**	**[0.82; 1.43]**	**100.0%**

Heterogeneity: I^2 = 37.0%, τ^2 = 0.0241, p = 0.2043

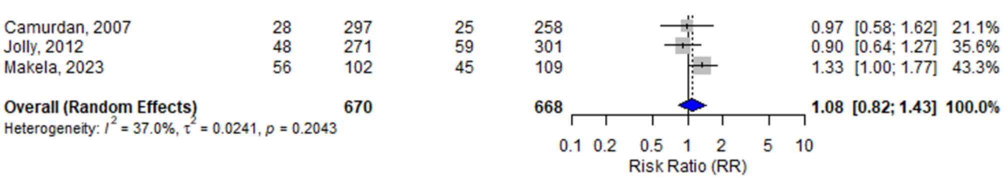

Figure S5: A random effect meta-analysis comparing the risk ratio of (A) less than four months of EBF in LMICs, (B) less than four months of EBF in HICs, (C) four months of EBF in LMICs, (D) four months of EBF in HICs, (E) six months of EBF in LMICs, and (F) six months of EBF in HICs among mother-infant pairs that received BFHI intervention with those that received only routine care.
